# Comparison of automatic and visual methods used for image segmentation in Endodontics: a microCT study

**DOI:** 10.1590/1678-7757-2017-0023

**Published:** 2017

**Authors:** Polyane Mazucatto Queiroz, Karla Rovaris, Gustavo Machado Santaella, Francisco Haiter, Deborah Queiroz Freitas

**Affiliations:** 1 Universidade Estadual de Campinas, Faculdade de Odontologia de Piracicaba, Departamento de Diagnóstico Oral, Área de Radiologia Oral, Piracicaba, SP, Brasil

**Keywords:** Dental pulp cavity, Threshold limit values, X-ray microtomography

## Abstract

To calculate root canal volume and surface area in microCT images, an image segmentation by selecting threshold values is required, which can be determined by visual or automatic methods. Visual determination is influenced by the operator's visual acuity, while the automatic method is done entirely by computer algorithms.

**Objective:**

To compare between visual and automatic segmentation, and to determine the influence of the operator's visual acuity on the reproducibility of root canal volume and area measurements.

**Material and methods:**

Images from 31 extracted human anterior teeth were scanned with a μCT scanner. Three experienced examiners performed visual image segmentation, and threshold values were recorded. Automatic segmentation was done using the “Automatic Threshold Tool” available in the dedicated software provided by the scanner's manufacturer. Volume and area measurements were performed using the threshold values determined both visually and automatically.

**Results:**

The paired Student's t-test showed no significant difference between visual and automatic segmentation methods regarding root canal volume measurements (p=0.93) and root canal surface (p=0.79).

**Conclusion:**

Although visual and automatic segmentation methods can be used to determine the threshold and calculate root canal volume and surface, the automatic method may be the most suitable for ensuring the reproducibility of threshold determination.

## Introduction

In Endodontics, it is important for some studies to have a morphometric analysis of teeth for evaluating aspects such as the shaping ability of endodontic instruments, simulated root canal abnormalities, decalcification and sectioning techniques. However, some techniques can have limitations that do not fulfill the requirements for some researchers^25^.

Microtomography is an imaging modality with increasing application in dental research due to its non-destructive technology that enables visualization of anatomical structures at the micrometer level^7^. In Endodontics, microtomography allows for qualitative and quantitative three-dimensional analyses of root canals while maintaining root integrity^12^ ^,^ ^17^ ^,^ ^21^ ^,^ ^22^ ^,^ ^28^. Also, the results obtained with this modality can be as good as those obtained with histological images for endodontic analyses^8^ ^,^ ^32^. Since the knowledge of the root canals’ anatomical subtleties is essential in Endodontics^6^ ^,^ ^31^, there have been several studies measuring root canal surface and volume by microtomography^1^ ^,^ ^3^ ^,^ ^10^ ^,^ ^12^ ^,^ ^13^ ^,^ ^21^ ^,^ ^25^ ^,^ ^27^.

To calculate root canal volume and surface area, one needs to begin with image segmentation. First, thresholding is applied to images, resulting in binarization (black and white). It is essential that the grey value thresholds for dental hard tissues and root canal spaces are carefully determined, as inadequacies in this step may result in an over- or underestimation of measurements^20^.

The correct inclusion of the region of interest in the binarized image is influenced by the choice of the segmentation threshold values used^8^. These values will determine what will be considered white and thus included in the analysis, or black and thus excluded from the analysis.

Thresholds can be determined by visual or automatic methods. Visual determination is influenced by the operator's visual acuity, which results in a subjectivity bias. To overcome this problem, an automatic threshold determination has been proposed. However, it is unclear whether differences between these segmentation methods are indeed significant in the context of microtomography studies. Studies generally use the visual method of image segmentation; however, the software in which the analysis is performed allows automatic segmentation, which is an option not commonly used to perform this step of the analysis of microtomographic images. Thus, this study was set to compare visual and automatic segmentation methods and to determine the influence of the operator's visual acuity on the reproducibility of root canal volume and area measurements.

## Material and methods

This research was approved by the local ethics committee (14905013.8.0000.5441/290.975).

Thirty-one extracted anterior and single-rooted maxillary and mandibular teeth with complete root formation, similar size and without any intracanal filling comprised the sample of this study. After chemical disinfection in a 2% glutaraldehyde solution for two hours, tooth crowns were sectioned near the cementoenamel junction, using a carborundum disc coupled to a metallographic cutter Isomet 1000^®^ (Buehler Ltd, Lake Bluff, IL, USA). A wax base was made as a support for each tooth.

**Figure 1:**
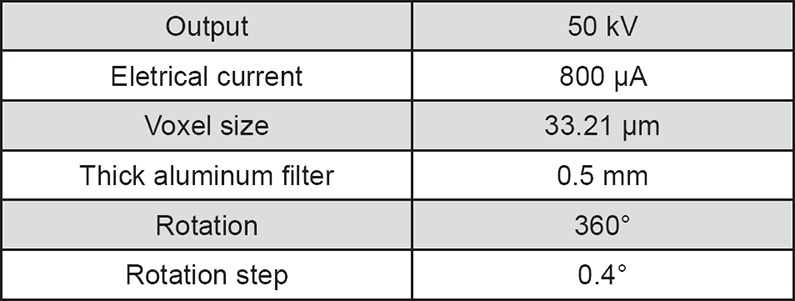
Scanning parameters

Images were captured with a SkyScan 1174 microCT unit (Bruker, Kontich, Belgium) and the scanning parameters are shown in Figure 1. After capturing, NRecon version 1.6.6.0 software (Bruker, Kontich, Belgium) was used for image reconstruction, applying a ring artefact correction (set at 4) and a 30% beam hardening correction.

Morphometric parameters were calculated with the CT-Analyzer software (Bruker, Kontich, Belgium). Slices starting from slightly coronal to the cementoenamel junction thru the apex were used to obtain measurements. Three experienced examiners, doctoral students in oral radiology with three years of experience in microtomography, after a calibration session for this analysis, performed visual image segmentation, and all threshold values were recorded. After 15 days, the images were re-evaluated, for manual and automatic methods. Automatic segmentation was done using the “Automatic Threshold Tool” available on CT-Analyzer as suggested by Otsu^15^ (1979). Three-dimensional analyses were performed using the values determined with the visual and the automatic method.

**Figure 2:**
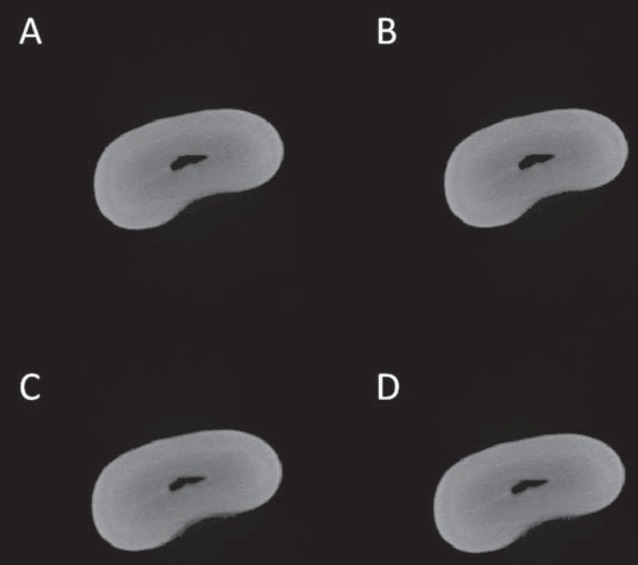
Axial slices of the root selected by different segmentation methods. A: automatic method; B: visual method – Examiner # 1; C: visual method – Examiner # 2; D: visual method – Examiner # 3

**Figure 3:**
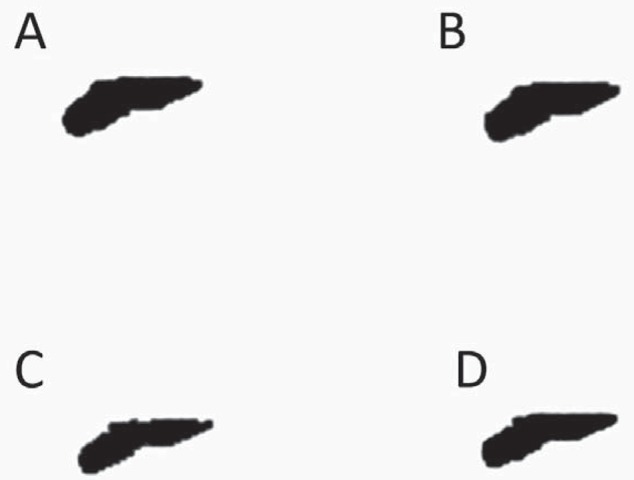
Axial slices of the unfilled cavity of root canal obtained by different segmentation methods. A: automatic method; B: visual method – Examiner # 1; C: visual method – Examiner # 2; D: visual method – Examiner # 3

Both visual and automatic segmentation (Figure 2) were applied to the root's dentin, since it is not possible to directly segment the root canal so that the canal is individualized as an unfilled cavity (Figure 3); instead, it maintains a shade of grey similar to that of the background. The same slices interval has been predetermined for each tooth, in which the first axial cut was determined immediately after the cementoenamel junction, and the last axial view of the tooth apex was determined as the last slice. After determination of the threshold limits for the root's dentin visually and automatically, it is necessary to use advanced tools (*i.e.*, custom processing) before proceeding with the measurements of canal volume and area. The seven steps of the sequence used for custom processing were: 1- Reload the image; 2- Threshold (fill in the threshold values already determined); 3- Despeckle (Remove pores – By image borders – 2D – Image); 4- Bitwise operations (Region of interest – Copy – Image); 5- Reload the image; 6- Threshold (same as step 2); 7- Bitwise operations (Image = Region of interest – Sub – Image).

**Figure 4:**
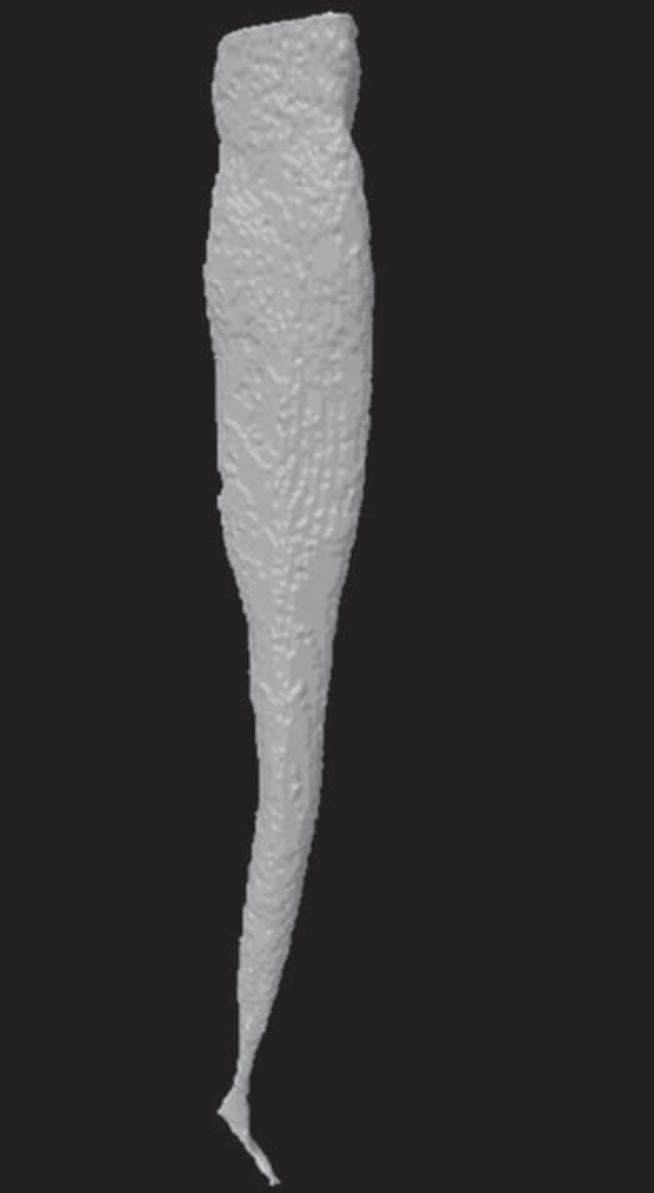
Image of a 3D model of the root canal after automatic segmentation

These steps were recorded and rerun for the other images. After processing, the resultant image should be representative of the morphology of the canal. However, other steps may be necessary with atresic canals. To create the 3D models (Figure 4), the processed images must be saved and loaded into the CTVox software (Bruker, Kontich, Belgium). Volume and area can be determined by loading the saved images into CT-Analyzer, then proceeding with segmentation of the canal's space; since the image is already binarized at this point, subjectivity is not an issue. After binarization, the images had only two colors: white, which is included in the analysis, and black (background), which is excluded. After that, one can perform the 3D analysis and obtain the volume and area of root canals.

Root canals’ volume and area for the thresholding values of each evaluator and for the automatic method were obtained for each tooth. The mean volume and area for the three evaluators were calculated, composing the visual method, and used for comparison with the automatic method. A paired Student's t-test was used to assess the influence of the methods on threshold determination and to identify any existing significant differences among the measurements obtained. The null hypothesis was that the method of threshold determination does not affect the measurement of root canal volume, considering an α=0.05. The Intraclass Correlation Coefficient (ICC) was calculated to evaluate intra- and inter-examiner agreement. Analyses were performed using MedCalc 15.8 (MedCalc Software, Ostend, Belgium).

## Results

The ICC for intra- and inter-examiner agreement for the visual method ranged from 0.47 to 0.914 and from 0.699 to 0.847, respectively, which is considered fair to excellent, according to Cicchetti^2^ (1994). For the automatic method, the ICC was of 1.0, showing a perfect correlation between the analyses.

Mean and standard deviation of canal volume and canal surface using different segmentation methods are shown in Table 1.

**Table 1:** Mean and standard deviation of canal volume and canal surface using different segmentation methods

	Canal volume (mm^3^)	Canal surface (mm^2^)
Automatic	2.85 (±1.29)^a^	26.43 (±6.19)^b^
Visual	2.88 (±1.26)^a^	26.95 (±8.26)^b^

Values followed by different letters (vertical) are significantly different (p<0.05)

There were no statistically significant differences between visual and automatic segmentation methods regarding root canal volume measurements (p=0.93) and root canal surface (p=0.79).

## Discussion

In Endodontics, microtomography has been extensively used as a research tool and a gold standard to test and compare endodontic files and to assess the anatomy of root canals. This imaging modality is used to evaluate three-dimensional images^18^ ^,^ ^28^, perform measurements^5^ ^,^ ^14^ ^,^ ^19^, allow for image segmentation to evaluate the structures of interest^11^ ^,^ ^23^ or to determine root canal volume^4^ ^,^ ^16^ ^,^ ^24^ ^,^ ^26^ ^,^ ^29^ ^,^ ^30^.

Dedicated software provided by the manufacturer of the microtomography unit is normally used to analyze the acquired images and determine the volume and area of root canals. However several steps are necessary to obtain that information; among them, image segmentation which can be achieved by visual or automatic methods, are indeed significant in the context of microtomography studies, because it is unclear whether there are differences between these segmentation methods. The image segmentation is a crucial phase for proper calculation of the volume and surface of a root canal. As microCT is a validated research method for root canal analyses^8^ ^,^ ^32^, this study proposed to evaluate if there are differences in the methods used to perform image segmentation.

Since no study carried out to compare the two methods of image segmentation was found in the literature, a direct comparison with our results was not possible. Researchers have done segmentation by visual and automatic methods without knowing if there are differences between them in the evaluation of the images. While some studies employed visual segmentation prior to root canal volume measurement^4^ ^,^ ^9^ ^,^ ^11^ ^,^ ^16^ ^,^ ^23^, others presented only the measurements without any description as to how image segmentation was performed^10^ ^,^ ^25^ ^,^ ^29^ ^,^ ^30^. Alternatively, few researchers used automatic segmentation before calculating root canal volume^26^.

The visual segmentation could be dependent on the examiner's sight and/or experience with image processing; thus, it is open to subjectivity. Still, the visual method allows the evaluator to control the segmentation process by determining the threshold from values of the segmented areas. Thus, it may be that for segmentation of more complex structures (for example, images of structures that present metal and, consequently, artifact), the visual method might allow a more accurate segmentation. However, in this study there were no differences in relation to automatic segmentation.

The automatic method is based on threshold determination from image histograms. It is carried out by the microtomography analysis software, which enhances reproducibility by leaving subjectivity out of the picture. Yet, it is worth mentioning that intraand inter-examiner agreement levels were lower when applying visual segmentation in comparison with the automatic method, which showed a perfect reproducibility. It is known that reproducibility represents the constancy of the method and is an important advantage of the technique. To determine agreement, we used measurements for volume and area with reasonable results, given that working with low values may overemphasize small differences among datasets. In addition, it might be easier and demand less time, since it is an automatic method. Thus, the automatic method is the most suitable for ensuring the reproducibility of threshold determination in endodontic studies. Nevertheless, further studies are needed to evaluate the reproducibility of segmentation using automatic threshold in other image segmentation tasks.

## Conclusion

Visual and automatic segmentation methods can be used to determine the threshold and to calculate root canal volume and surface; however, the automatic method may be the most suitable for ensuring the reproducibility of threshold determination. It is up to the evaluator to choose the preferred method according to their experience and the available time to perform image segmentation.
